# *Helicobacter pylori* in patients with gastritis in West Cameroon: prevalence and risk factors for infection

**DOI:** 10.1186/s13104-018-3662-5

**Published:** 2018-08-03

**Authors:** Nathan E. Agbor, Seraphine N. Esemu, Lucy M. Ndip, Nicoline F. Tanih, Stella I. Smith, Roland N. Ndip

**Affiliations:** 1 Department of Microbiology and Parasitology, University of Buea, P. O. Box 63, Buea, Cameroon; 2 Laboratory for Emerging Infectious Diseases, University of Buea, Buea, Cameroon; 3 Medical Research Council Unit, Fajara, Gambia; 4 Nigerian Institute of Medical Research, Lagos, Nigeria

**Keywords:** Gastritis, *Helicobacter pylori*, Prevalence, Risk factors, Cameroon

## Abstract

**Objectives:**

*Helicobacter pylori* is a pathogenic bacterium that parasitizes the gastric mucous layer and the epithelial lining of the stomach causing duodenal ulcers, gastric ulcers and cardiovascular disease amongst others. This study aimed at establishing the epidemiologic profile of *H*. *pylori* infection in gastritis patients presenting at the Melong District Hospital.

**Results:**

Blood, stool and epidemiological data collected from 500 patients were analyzed for the presence of *H. pylori* antibody in serum, antigen in stool and elucidation of risk factors captured in questionnaires. Of 500 blood samples, 217 (43.4%) were seropositive with male and female seroprevalences of 45.5% (61/134) and 42.6% (156/366) respectively. Similarly, 47.4% (237/500) samples tested positive for stool antigen with prevalences of 47.0% (63/134) for males and 47.5% (174/366) for females. The antigen prevalence was higher (53.2%; 118/222) in older patients (> 50 years) than in younger patients (42.8%; 119/278; P = 0.021). The antigen test had a higher (47.4%) prevalence than the antibody test (43.4%). Educational level, source of income, source of drinking water, age of patients, and alcohol consumption had positive associations with *H*. *pylori* infection. These results have clinical and epidemiological significance and call for intervention to mitigate the situation.

## Introduction

*Helicobacter pylori,* a bacterium that parasitizes the gastric mucous layer and the epithelial lining of the stomach, is a Class I carcinogen and the predominant bacterium that colonizes the stomach mostly during childhood [1, 2]. This bacterium infects over 50% of the world’s population and is a frequent cause of chronic bacterial infections [3]. Approximately 10% of infected individuals develop overt clinical disease while 90% remain subclinical and the infection can persist throughout life if untreated [4]. So far, *H. pylori* has 20 recognized strains [5] that have been implicated in many diseases including duodenal ulcers, gastric ulcers, adenocarcinoma of the distal stomach, mucosa-associated lymphoid tissue (MALT) lymphoma, diabetes mellitus, cardiovascular disease and autoimmune disease [1, 5]. Treatment failure of *H. pylori* infection has been linked to bacterial resistance and poor patient compliance [6, 7].

The routes of transmission of *H. pylori* have not been clearly identified. Houseflies have been shown to have the potential to transmit the organism mechanically implying poor sanitation is a risk factor for its spread [8]. Transmission from person-to-person and through tubes or endoscopes have been reported. Use of water contaminated with faeces may predispose people to *H*. *pylori* infection [8, 9] and if the water source is a municipal water supply, this may potentiate its spread. Factors that have been associated with the acquisition of *H. pylori* infection include high density crowding, poor sanitary practices, family income, educational level, age, occupation, religion and poor water supply [4, 10, 11].

There is great geographical variation in the prevalence of *H. pylori* with higher prevalence in developing than in developed countries [1, 12]. Knowledge of *H. pylori* status is very important in patient management. Diagnosis relies on invasive and non-invasive techniques and non-invasive techniques (such as stool test and serology) are preferable in epidemiological studies [13]. The stool test demonstrates the presence of antigens while serology detects antibodies to *H*. *pylori*. However, serologic tests are limited by false positivity because of cross-reactions [13]. Previous studies have confirmed the superiority of the stool antigen test over serology in terms of true outcomes and cost [14].

There is a paucity of information on the prevalence of *H*. *pylori* and associated risk factors in Cameroon. Previous studies in Cameroon reported *H. pylori* prevalence of 72% (67/93) from biopsies samples with evidence of gastritis [15] and 92.2% from gastric biopsies of patients with gastroduodenal pathologies [16]. It was therefore important to conduct this hospital-based cross-sectional study to generate recent spatiotemporal data on *H*. *pylori* prevalence and risk factors for infection to inform prevention and control measures.

## Main text

### Study site and participants

The study was conducted at the Melong District Hospital which serves as a referral centre for health facilities within the Melong Health District. Melong is located at 5°07′18″ N and 9°57′41″ E in a rainforest zone and 792 m above sea level. The inhabitants are predominantly traders, artisans and farmers and their socioeconomic status predisposes them to infections associated with personal and environmental hygiene. Five hundred patients of both sexes, who consulted at the hospital with signs and symptoms of chronic gastritis, were enrolled in this study from August 2013 to September 2015.

### Ethical considerations

Ethical clearance was obtained from the Institutional Review Board of the Faculty of Health Sciences, University of Buea (Reference No. 2015/347/UB/FHS/IRB of 1 July 2015). Administrative authorizations were obtained from the Regional Delegation of Public Health for the Littoral Region and the Director of the Melong District Hospital. Samples were collected only from patients who gave their consent to participate in the study.

### Sample collection

Blood and stool were collected for serology and antigen tests respectively. Epidemiological data were also collected for risk factors assessment. Two milliliters of blood were collected aseptically from each study participant by venipuncture and transferred to a sterile vacutainer tube without anticoagulant. The blood was allowed to clot by leaving it undisturbed at room temperature for 45 min, centrifuged at 2000×*g* for 10 min and the serum harvested. The stool sample (1–2 mL or 1–2 g) was collected with the use of a catching device and put in a sterile screw-capped bottle. All samples were stored at + 4 °C until processed.

Epidemiological data captured on questionnaires included socio-demographic data (sex, age, marital status, educational level), socioeconomic factors (household population, source of income, source of drinking water and toileting system) and health determining behaviours (cigarette and alcohol consumption).

### Sample processing

Serum analysis was done using a One-step *H. pylori* antibody test device (DiaSpot, Indonesia) according to the manufacturer’s instructions. Briefly, three drops of the serum sample were applied directly into the sample well in the test device and results read after 10 min. The appearance of one coloured line on the control region indicated a negative result while the appearance of two coloured lines on the test region and control regions indicated positive result.

Stool analysis was done using a One-step *H. pylori* antigen test device (ABON Bioparm Hangzhou, China). The test was performed according to the manufacturer’s instructions without any modifications. Briefly, 50 mg (from formed stool) or two drops of liquid stool were transferred to a specimen collection tube containing extraction buffer. The tube was agitated vigorously by hand-shaking and left undisturbed for 2 min. Two drops of the extracted specimen were transferred to the specimen well on the test device and results read after 10 min.

### Statistical analysis

The χ^**2**^ test was used to analyze the data. *P *< 0.05 was considered statistically significant. All calculations were carried out with Epi Info version 3.5.4 (CDC, Atlanta, USA).

## Results

### Prevalence of *H. pylori* infection

Of the 500 stool samples analysed, *H*. *pylori* antigen was detected in 237 (47.4%). The prevalence of *H*. *pylori* antigen in females was 47.5% (174/366) and 47.0% (63/134) in males. However, this difference was not statistically significant. The distribution of the *H*. *pylori* stool antigen positive samples with respect to other patient characteristics are presented in Table 1. Overall, 217 (43.4%) of the 500 serum samples analysed had antibodies against *H*. *pylori*. Similarly, the seroprevalence in females (42.6%, 156/366) was almost the same as that in males (45.5%, 61/134). Most (56.7%, 123/217) of the seropositive samples were from patients less than 50 years old (Table 1).

**Table 1:** Characteristics of study participants and *Helicobacter pylori* positivity

Variables	Total participants	Number positive for *H*. *pylori*		P-value
		Stool antigen (%)	Serology (%)	
Sex				
Male	134	63 (47.0)	61 (45.5)	0.917
Female	366	174 (47.5)	156 (42.6)	
Age group (years)				
< 50	278	119 (42.8)	123 (44.2)	0.021**
> 50	222	118 (53.2)	94 (42.3)	
Education				
None	25	20 (80.0)	18 (72.0)	0.000**
Primary	235	125 (53.2)	111 (47.2)	
Secondary	207	92 (44.4)	88 (42.5)	
Tertiary	33	0 (0)	0 (0)	
Source of income				
Agriculture/trading	360	177 (49.2)	160 (44.4)	0.000**
Service	52	22 (42.3)	21 (40.4)	
Housewife/retired	68	20 (29.4)	20 (29.4)	
Others	20	18 (90.0)	16 (80.0)	
Source of drinking water				
Tapwater	240	105 (43.8)	98 (40.8)	0.006**
Spring	250	125 (50.0)	113 (45.2)	
Bottled	7	7 (100)	6 (85.7)	
Others	3	0 (0)	0 (0)	
Duration of gastritis symptoms (years)				
< 1	197	74 (37.6)	70 (35.5)	0.003**
1–3	110	60 (54.5)	57 (51.8)	
3–4	47	22 (46.8)	15 (31.9)	
> 4	146	81 (55.5)	75 (51.4)	
Household population				
1–3	142	69 (48.6)	61 (43.0)	0.055
4–5	220	108 (49.1)	97 (44.1)	
> 5	138	60 (43.5)	59 (42.8)	
Toileting system				
Leads to drainage	122	64 (52.5)	60 (49.2)	0.044**
Closed pit	365	167 (45.8)	153 (41.9)	
Others	13	6 (46.2)	4 (30.8)	
Alcohol consumption				
Yes	332	169 (50.9)	156 (47.0)	0.027**
No	168	68 (40.5)	61 (36.3)	
Tobacco consumption (smoker)				
Yes	132	59 (44.7)	57 (43.2)	0.468
No	368	178 (48.4)	160 (43.5)	
Smokers (n = 132)				
Regular smokers	78	25 (32.1)	22 (28.2)	0.000**
Occasional smokers	54	34 (63.0)	29 (53.7)	

Stool antigen test considered as gold standard

** Significant differences

The antigen test detected a higher number (237) of positive samples and was used to calculate the sensitivity and specificity of the antibody test which were 89.9 and 98.5% respectively. The antibody test failed to detect 24 samples which were positive for *H. pylori* using antigen test. Four samples were positive by the antibody test only. Test results were concordant for 213 samples. A total of 24 samples produced discordant results (Table 2).

**Table 2:** Outcome of the detection of *H*. *pylori* infection in 500 patients by both tests

	Stool antigen test			Total
		+	–	
Serology	+	213	4	217
	–	24	259	283
Total		237	263	500

Although the antigen test consistently detected a higher number of positives in all sub-populations of the patients analysed (Table 1) The difference in the detection rates between the two tests was not significant (P = 0.204) (Table 2).

### Risk factors for *H*. *pylori* infection

This study revealed several risk factors for *H*. *pylori* infection. Patient variables, including socioeconomic characteristics, were examined for associations with *H*. *pylori* infection (Table 1). Education, age, source of income, source of drinking water, alcohol consumption and toileting system had statistically significant (P < 0.05) associations with *H. pylori* infection. The prevalence of the infection decreased with increasing level of education with highest (20/25, 80%) prevalence among those with no level of education and lowest (0%) prevalence among those who had tertiary level of education. Tobacco consumption, household population, and sex showed no association (P > 0.05) with *H. pylori* infection.

## Discussion

This study sought to establish the epidemiological profile of *H. pylori* in patients presenting at the Melong District Hospital with signs and symptoms of gastritis. The large representation (73.2%, 366/500) of females in the study population revealed that more females consulted at the hospital with symptoms of chronic gastritis than males (26.8%, 134/500). This large representation of females could be due to the global demographic profile which shows that the women population exceeds that of the men. However, there was no statistically significant difference in the antigen prevalence in males and females corroborating earlier results [17].

The prevalence of *H*. *pylori* has a wide geographical variation and over the years, decreasing prevalence has been reported in several areas of the globe [1, 18]. The overall prevalence of *H. pylori* infection in this study was 43.4% (217/500) for serology and 47.4% for stool antigen test. A previous study reported a seroprevalence of 36.7% in rural Vietnam [19]. Generally, the prevalence of *H. pylori* is expected to decrease with improvement in the socioeconomic and hygiene status of the population. An earlier study reported an *H. pylori* seroprevalence of 66.9% in 1998 which dropped to 59.6% in 2005 in South Korea [20]. *H. pylori* prevalence varies from one place to another based on the socioeconomic and hygienic condition of the environment and in the same environment, the prevalence of the same infection can vary with time [20]. There was no statistically significant difference (P = 0.917) in the seroprevalence results between females (42.6%, 156/366) and males (45.5%, 61/134) and similar results have been reported elsewhere [17]. The antigen prevalence (47.4%) reported in this study was low compared to that reported earlier (52.27%; 92/176) in stool of asymptomatic children in Buea and Limbe health districts of Cameroon [21]. While an earlier study in Cameroon reported a high *H. pylori* prevalence (92.2%, 71/77) in patients referred for endoscopy [16], a study in the North West Province of Cameroon showed a lower prevalence of 72% (67/93) [15]. These differences in prevalence may be due to improvement in the socioeconomic and hygiene conditions of the population over time. In a recent study carried out in North Sulawesi and Indonesia, a much lower prevalence of 14.3% (36/251) was reported [18].

Several studies have been published on risk factors for infection, but the findings have been conflicting. Generally, the infection has been shown to be higher among those with low socioeconomic and hygiene state [22]. The antigen prevalence in elderly persons (≥ 50 years) was higher (53.2%) than in the young (< 50 years) (42.8%) and this difference was statistically significant (P < 0.05). Previous studies also noted that the infected participants were significantly older than those that did not have the infection [17, 23]. Ageing is associated with a diminished epithelial cell turnover rate and a reduced capacity to repair the gastric mucosa [23] and this has been attributed to decreasing prostaglandin levels in the gastric mucosa making age a major risk factor for *H. pylori* colonization [24].

Studies on association between *H. pylori* and smoking or alcohol drinking report conflicting results. A study in Brazil reported no statistical significant effect of smoking and alcohol consumption on *H. pylori* prevalence [25]. Our study showed that *H. pylori* infection was higher among alcohol consumers (51.1%) than among those who had never had alcohol consumption as a habit (40.3%) and this corroborates a previous study [26].

The antigen prevalence was lower among smokers (44.7%, 59/132) than among non-smokers (48.4%, 178/368). Similarly, regular smokers had a lower prevalence of 32.1% compared to 63.0% among the occasional smokers. This contradicts a previous study that reported that *H. pylori* infection was more in smokers and ex-smokers than in those who had never smoked [27]. An earlier attempt to study these risk factors could not establish any relationship between the infection and smoking [17]. In a recent large scale study, it was reported that smoking was negatively associated with *H. pylori* infection and the risk of the infection was noted to linearly decrease with cigarette consumption per day [28]. Cigarette smoking results in increase in gastric acidity and this may account for the negative association between smoking and *H. pylori* infection [29].

## Conclusion

This study revealed an *H. pylori* seroprevalence and antigen prevalence of 43.4 and 47.5% respectively with no statistically significant difference. Among risk factors assessed, educational level, source of income, cigarette smoking and alcohol consumption were statistically associated with *H. pylori* infection.

### Limitations

All individuals sampled were hospital patients with signs and symptoms of chronic gastritis hence the spatiotemporal results from this study cannot be generalized to represent *H*. *pylori* prevalence and risk factors in the general population in Cameroon. This study needs to be expanded to other areas of the country as well as the inclusion of healthy individuals as a control group.

## Abbreviations

*H*. *Pylori*: *Helicobacter pylori*
MALT: mucosa-associated lymphoid tissue
